# Functional Role of Adult Hippocampal Neurogenesis as a Therapeutic Strategy for Mental Disorders

**DOI:** 10.1155/2012/854285

**Published:** 2012-12-31

**Authors:** Heechul Jun, Syed Mohammed Qasim Hussaini, Michael J. Rigby, Mi-Hyeon Jang

**Affiliations:** ^1^Department of Neurologic Surgery, Mayo College of Medicine, 200 First Street SW, Rochester, MN 55905, USA; ^2^Summer Undergraduate Research Fellowship Program, Mayo Graduate School, Mayo College of Medicine, 200 First Street SW, Rochester, MN 55905, USA; ^3^Department of Biochemistry and Molecular Biology, Mayo College of Medicine, 200 First Street SW, Rochester, MN 55905, USA

## Abstract

Adult neurogenesis, the process of generating new neurons from neural stem cells, plays significant roles in synaptic plasticity, memory, and mood regulation. In the mammalian brain, it continues to occur well into adulthood in discrete regions, namely, the hippocampus and olfactory bulb. During the past decade, significant progress has been made in understanding the mechanisms regulating adult hippocampal neurogenesis and its role in the etiology of mental disorders. In addition, adult hippocampal neurogenesis is highly correlated with the remission of the antidepressant effect. In this paper, we discuss three major psychiatric disorders, depression, schizophrenia, and drug addiction, in light of preclinical evidence used in establishing the neurobiological significance of adult neurogenesis. We interpret the significance of these results and pose questions that remain unanswered. Potential treatments which include electroconvulsive therapy, deep brain stimulation, chemical antidepressants, and exercise therapy are discussed. While consensus lacks on specific mechanisms, we highlight evidence which indicates that these treatments may function via an increase in neural progenitor proliferation and changes to the hippocampal circuitry. Establishing a significant role of adult neurogenesis in the pathogenicity of psychiatric disorders may hold the key to potential strategies toward effective treatment.

## 1. Introduction 

Mental disorders are debilitating conditions that significantly impair the function of the central nervous system and degrade the quality of life. About one-quarter of adult Americans are diagnosed with mental disorders such as major depression, anxiety, and schizophrenia [1]. Understanding the neurobiological basis of mental disorders, determining effective treatments, and alleviating the respective symptoms are major forces driving modern psychiatry today. 

The hippocampus, an area of the brain important in memory, cognitive function, and mood regulation, is particularly vulnerable to chronic stress and mental disorders [2, 3]. Several landmark clinical studies have demonstrated that major depression is accompanied by a decrease in the volume of hippocampus and consequent deficits in hippocampal function [4, 5]. Similarly, in schizophrenic patients, shape deformations, cell loss, and volume reduction in the hippocampus were found using neuroimaging analysis [6–8]. Reversal of these alterations has successfully improved the behavioral and cognitive symptoms associated with these disorders. Such evidence has encouraged consideration of whether improving hippocampal structure and function could be a potential therapeutic target in treating mental disorders [9, 10].

Since the pioneering discovery of mammalian postnatal neurogenesis in the 1960s [58], adult neurogenesis has been unambiguously investigated in discrete brain regions across mammals including humans. Adult neurogenesis in all mammals, including humans, occurs throughout life within two specialized neurogenic niches, the subventricular zone (SVZ) of the lateral ventricle and the subgranular zone (SGZ) of the hippocampal dentate gyrus [59]. In particular, adult neurogenesis in dentate gyrus has attracted interest since newborn neurons contribute to enhanced neural plasticity that could sustain specific brain functions such as spatial learning, pattern discrimination, and mood regulation [47, 60, 61]. In addition, adult hippocampal neurogenesis in the mature brain represents a striking example of activity-dependent neural plasticity such as stress, antidepressants, and brain injuries [62]. Extensive studies have shown that voluntary exercise, enriched environments, and antidepressants contribute to overall brain health by robustly promoting adult hippocampal neurogenesis [37, 53, 63]. Decreased neurogenesis in the hippocampus via aging or stress has been implicated in the pathogenesis of cognitive deficits, anxiety and depression [64, 65]. In fact, adult hippocampal neurogenesis not only plays an important role in antidepressant action [47] but also plays a role in ameliorating various pathological disease conditions [25, 46, 66, 67]. Therefore, a better understanding of the molecular and cellular mechanisms that regulate adult hippocampal neurogenesis may offer new therapeutic targets. 

In this paper, we will highlight three major psychiatric disorders that have been associated with adult hippocampal neurogenesis. We will present and interpret the significance of the results in regards to the mechanism of cognitive and neurological disorders. Finally, we will lay out some current and potential therapeutic treatments that are used to counter these psychiatric disorders.

## 2. Adult Neurogenesis and Mental Disorders

### 2.1. Depression

Major depression is among the most prevalent psychiatric disorders and has high morbidity worldwide. Chronic stress represents a key risk factor in developing depression [68–70]. Despite a tremendous amount of study, the underlying mechanisms associated with the pathophysiology of depression remain poorly understood. The neurogenesis hypothesis of depression originated from studies using animal stress models. Because of the lack of a pathophysiologically reliable animal depression model, stress is primarily used to cause depression in animal models. These studies demonstrate that stress inhibits newborn neuron proliferation in the dentate gyrus of the hippocampus [71, 72]. All antidepressant classes are now known to promote adult hippocampal neurogenesis [38, 47]. The underlying cellular and molecular mechanisms of adult neurogenesis in regulating the suppressive effect of stress have been examined using various stress paradigms including physical and psychosocial stresses (Table 1). For example, physical stress such as repeated restraint [11] and inescapable foot shock [12] inhibits one or more steps of adult neurogenesis in the dentate gyrus. Similarly, chronic psychosocial stress using the social defeat paradigm leads to an inhibitory effect on cell proliferation and survival of newborn granule neurons in rodents [17]. In addition, the effect of depression on hippocampal volume in humans has been studied [4, 5, 73–75]. Early onset depression has been associated with a reduction in hippocampal volume [75], and in patients older than 60, depression has been associated specifically with a reduction in right hippocampal volume [74]. Further, hippocampal grey matter which undergoes reduction in depressed patients could be increased through antidepressive treatment like citalopram [73]. While such changes have been observed, postmortem studies have found no change in cell proliferation between major depression patients and control samples [76, 77]. Intriguingly, antidepressant treatment does increase cell proliferation in the dentate gyrus of major depression patients [77]. Such differential effects between rodents and humans on cell proliferation in the dentate gyrus might be due to biological differences implying different cellular mechanisms [78]. Determining the processes of adult neurogenesis in humans, such as fate determination, survival, differentiation, and integration across both spectrums will be helpful in reconciling the differences.

Preclinical models of depression generally show one or more suppressed steps during the sequential adult neurogenesis process. Such evidence has raised questions regarding the causality between neurogenesis and depression. Using a variety of methods ablating newborn neurons in the adult dentate gyrus, evidence correlates adult hippocampal neurogenesis with depression. However, the results are controversial [47, 48, 76, 79]. For example, ablating adult hippocampal neurogenesis using X-ray irradiation does not affect anxiety- and depressive-like behaviors as measured by novelty-suppressed feeding, open-field, light-dark, and elevated plus maze [47, 48, 79]. Further, pharmacological treatment with an anti-mitotic drug, methylazoxymethanol (MAM) which decreases cell proliferation in the dentate gyrus, does not induce an anhedonic-like state in rats [80]. On the contrary, genetically inhibiting hippocampal neurogenesis using Nestin-rtTA/Tet-Bax bigenic mice does increase anxiety-related behavior but does not affect depressive-like behavior [81]. Also, deletion of adult neurogenesis using GFAP-TK mice influences depressive-like behavioral response as shown by increased immobility time in the tail suspension test and induction of an anhedonic-like response in sucrose-preference test, but shows no effect in novelty-suppressed feeding or elevated plus maze [82]. This group further suggests that suppression of neurogenesis predisposes the animal to stress-induced anxiety/depression-like behavior response and that newborn neurons in the hippocampus buffer this depressive-like behavior [82]. At this juncture, the results do not quite support that decreased neurogenesis is associated with a risk factor in development of depressive behavior. It may be more pertinent to conclude that newborn neurons may be a major contributor in normalizing or ameliorating against disease state rather than being causally involved in the etiology of depression in the animal model. 

### 2.2. Schizophrenia

Schizophrenia is a multifactorial psychiatric disorder resulting from a complex interplay of genetic and environmental susceptibility factors [3]. Establishing the major etiology, neuropathology and pathophysiology of schizophrenia have proven difficult. 

Initial studies from postmortem hippocampal tissue have indicated a reduction in neural progenitors and hippocampal volume in patients diagnosed with schizophrenia [76]. Supporting this, a growing number of studies have identified susceptibility genes associated with schizophrenia that are involved in regulation of adult neurogenesis [22, 83] (Table 2). One such gene is disrupted-in-schizophrenia 1 (DISC1) which was originally identified as a potential susceptibility gene for schizophrenia and related psychiatric disorders in a large Scottish pedigree [84, 85]. It is widely expressed during embryonic neurogenesis and postnatal development with high expression persisting in the adult hippocampus, especially in the dentate gyrus [86, 87]. The expression pattern of DISC1 may implicate a role of DISC1 in neuronal development. Using various approaches including genetic mutants and short-hairpin RNA (shRNA) knockdown, several lines of evidence have converged to indicate that DISC1 function is involved in distinct steps of adult neurogenesis and behavioral response. First, mutant DISC1 mice with selective impairment in working memory showed reduced number of neural progenitors and immature neurons, as well as misoriented apical dendrites of immature neurons in the adult hippocampal dentate gyrus [21]. In the same DISC1 mutant mice, altered axonal targeting and short-term plasticity in the hippocampus were also observed [21]. Second, knockdown of DISC1 via lentiviral-mediated shRNA also led to decreased newborn cells and a depressive-like behavioral response [22]. Third, knockdown of DISC1 via retroviral-mediated shRNA approach to label dividing cells and their progeny results in aberrant dendritic development including an enhancement of dendritic outgrowth, soma hypertrophy, overextended migration, accelerated synapse formation, mistargeted axonal mossy fibers, and presynaptic differentiation of newborn granule neurons in the adult mouse dentate gyrus [18, 19]. Further, the same group also indicated that AKT-mTOR signaling pathway is a critical DISC1 target in regulating dendritic development of newborn granule neurons in the adult dentate gyrus [88]. Taken together, these results suggest that DISC1 orchestrates the tempo of functional integration in the adult brain. It also demonstrates the major roles a susceptibility gene could play in neuronal development and the pathogenesis of the disease.

Other susceptibility genes for schizophrenia linked to adult neurogenesis have been identified. NPAS, a bHLH transcription factor that is broadly expressed in the developing neuroepithelium, was identified as a risk factor for schizophrenia and associated with cognitive deficits in an affected mother and her daughter [89, 90]. Mice lacking *Npas3* show developmental brain abnormalities including a reduction in size of the anterior hippocampus, hypoplasia of the corpus callosum and enlargement of the ventricles [23]. In addition, mice lacking *Npas3* exhibit behavioral abnormalities including locomotor hyperactivity, subtle gait defects, impairment of prepulse inhibition of acoustic startle, deficit in recognition memory, and altered anxiety-related responses [23]. Finally, Npas3-deficent mice also display a significant reduction of adult neurogenesis by 84% relative to their wild-type littermates, which may suggest that adult neurogenesis impairment is involved in the pathogenesis of schizophrenia [22]. However, direct causal evidence linking adult neurogenesis to schizophrenia needs to be examined. 

In addition to using genetic animal models for schizophrenia, a prenatal injection of synthetic double-stranded RNA polyriboinosinic polyribocytidylic acid (PolyI:C) into mice has been used as an animal model for schizophrenia [26, 91]. These mice displayed behavioral deficits in the open-field test and prepulse inhibition of the startle response [26]. A recent study demonstrated that these infected animals showed behavioral impairments accompanied by decreased adult hippocampal neurogenesis [25]. Interestingly, these abnormalities were rescued by increasing adult hippocampal neurogenesis via exercise, indicating that enhancing neurogenesis may help aid recovery for schizophrenia [25].

### 2.3. Drug Addiction

Drug addiction is a chronic relapsing disorder characterized by a loss of the ability to control drug intake and a compulsive drug-seeking and -taking behavior [92]. Generally, the mesolimbic dopaminergic system in the brain is thought to be an important brain area in the neurobiology of addiction [93]. The hippocampus has received great attention because abusive drugs are potent negative regulators of adult hippocampal neurogenesis and as a result may impair cognitive function [27, 94–96] (Table 3). 

Cocaine abuse, a powerful and addictive psychostimulant drug, is associated with dynamic regulation of adult neurogenesis in the hippocampal dentate gyrus and its corresponding memory function [30]. Studies have shown that both a high dose of cocaine or chronic cocaine exposure can decrease the proliferation of neural progenitors in the rat dentate gyrus [28] and cause working memory dysfunction [30]. In addition, chronic treatment of cocaine increased adult neurogenesis in mice in some studies [97], but the same effect is not observed in the rat dentate gyrus [27, 31, 98, 99]. While most studies support evidence that chronic administration of cocaine diminishes proliferation of neural progenitors in the adult hippocampus, the effect on survival and maturation of neural progenitors is not always consistent. Possible explanations for the discrepancy might be due to the differences in animal strain, methods, and duration of cocaine treatment. Therefore, more evidence is needed to determine the timing, duration and consequences of impaired adult hippocampal neurogenesis caused by cocaine. Recently, role of adult hippocampal neurogenesis in cocaine-taking and cocaine-seeking behavior was explored. Using X-ray irradiation approach, ablation of adult hippocampal neurogenesis increased cocaine-taking and cocaine-seeking behavior, suggesting that impaired neurogenesis may result in increased susceptibility in the animal model of cocaine addiction [100]. Interestingly, withdrawal from cocaine administration normalizes the reduction of neural progenitors and enhances maturation of neural progenitors in the adult dentate gyrus [27]. This indicates that the normalization of the cocaine-induced neurogenesis deficit may help decrease susceptibility to relapse and related cognitive deficits. 

The correlation between alcohol dependence and hippocampal neurogenesis has been extensively studied. Among other anatomic changes, alcoholic patients undergo structural changes in hippocampal volume [101–103]. Animal models have been used to examine the cellular effects and underlying mechanisms. Converging studies have associated alcohol consumption and dependence with a selective decline in adult hippocampal neurogenesis [32, 104–107]. Acute or chronic administration of ethanol treatment in mice inhibits neural progenitor cell proliferation and survival in the adult rat hippocampus [32, 108]. Clinically relevant animal models have been used to confirm this wherein alcohol dependence reduced proliferation of neural progenitors, as well as consequent differentiation and maturation [34]. More recently, chronic alcohol treatment over 11 months in adolescent macaque monkeys produced selective and long-lasting decrease in hippocampal neural progenitor proliferation [36]. Alterations in synaptic plasticity are also associated with chronic alcohol treatment resulting in the reversible inhibition of long-term potentiation (LTP) in the rat hippocampus [109]. Besides LTP changes, functional or behavioral changes as measured via active avoidance, and spatial memory also occur following alcohol consumption indicating learning and memory deficits [110, 111]. An abstinence state following alcohol consumption can contribute to depression-like behavior with a concurrent reduction in the neural progenitor and immature neuron population in dentate gyrus in mice [35]. This was counteracted via desipramine which alleviated both the structural and functional phenotypes associated with this comorbidity [35]. Whether adult neurogenesis can regulate alcohol-drinking or -seeking behavior remains to be studied. Understanding this causality will allow us to develop improved therapeutic intervention in treating the pathological symptoms associated with alcohol dependence.

## 3. Therapeutic Interventions

Adult neurogenesis is affected by a variety of external stimuli that influence neuronal activity. The therapeutic effects of electroconvulsive therapy, antidepressants, exercise, and others such as deep brain stimulation have been utilized for their therapeutic efficacy. These have been associated with adult hippocampal neurogenesis and will be discussed below.

### 3.1. Electroconvulsive Therapy and Deep Brain Stimulation

One of the more intense therapeutic treatments for severe psychiatric disorders, especially depression, is electroconvulsive therapy (ECT) [112]. In the 1940s and 50s, ECT was used in extreme cases in humans when patients did not respond to other treatments [112, 113]. Despite extensive research, the mechanism of action of ECT had not been discovered. Recently, it was linked to neurogenesis, providing a possible explanation for the mechanism that helps to relieve symptoms [37, 38] (Table 4). 

The first study to demonstrate the influence of ECT on adult hippocampal neurogenesis showed that a single treatment of ECT results in stimulation of adult neural progenitors that survive up to 3 months afterward [37]. A subsequent study confirmed that ECT promotes neural progenitor proliferation [38]. Further, ECT also reversed long-term neurogenesis deficits and hippocampal-dependent fear memory disrupted by X-ray irradiation [114]. This matches clinical observations that ECT is the most effective treatment for depression. It may also suggest that adult hippocampal neurogenesis is a critical neurobiological component underlying the clinical effect of ECT. A detailed characterization of hippocampal progenitors affected by ECT has been reported. At the cellular level, ECT stimulates proliferation of quiescent progenitor cells and at a later phase increases the proliferation of amplifying progenitor cells [39], which may lead to a net increase in the number of mature adult-born neurons [40]. Functionally, electrophysiological analysis shows that chronic treatment of ECT induces long-term-potentiation-(LTP-) like synaptic changes in the adult dentate gyrus [115]. Given that LTP results in increased proliferation of neural progenitors in the dentate gyrus [116], enhanced neurogenesis caused by ECT could potentially alter hippocampal circuitry which may contribute to the functional effects of ECT. 

Exactly how ECT stimulates specific niche signals to regulate the sequential process of adult neurogenesis remains unclear. Growing evidence in the past decade has determined a number of factors that regulate adult neurogenesis in response to ECT in the hippocampus. Among those factors, induction of neurotrophic growth factors has been extensively identified. These include brain-derived neurotrophic factor (BDNF), fibroblast growth factor-2 (FGF-2), and vascular endothelial growth factor (VEGF) [105, 106, 117]. BDNF, a member of the nerve growth factor family expressed throughout the brain, is known to be responsible for synaptic strength, survival, and growth of mature neurons via activation of its receptor TrkB. While both acute and chronic treatments of ECT induce BDNF and TrkB gene expression, the level of BDNF gene expression remains prolonged during chronic treatment of ECT [106]. FGF family of growth factors and corresponding receptors are involved in angiogenesis and early stages of neural development [118, 119]. Expression of FGF-2 mRNA in rodent hippocampus is increased in both acute and chronic treatments of ECT [117]. In addition, infusion of BDNF and FGF into rodent hippocampus results in antidepressant-like effects [120, 121], while blockade of BDNF and FGF in the dentate gyrus induces behavioral deficits and decreases adult hippocampal neurogenesis [121, 122]. These findings indicate that such factors can be strong candidates in ECT treatment to mediate antidepressant effects. A recent study demonstrated ECT promoting DNA demethylation in the BDNF and FGF promoter regions and adult neurogenesis in dentate gyrus through growth arrest and DNA-damage-inducible protein 45 beta (Gadd45b) [42]. These results suggest that dynamic epigenetic DNA modifications may serve as an essential mechanism to translate neurogenic niche signals for sustained regulation of adult neurogenesis and antidepressant action of ECT. Lastly, VEGF is known as a regulator for vascular growth and also a stimulator of neurogenesis [123]. The effect of ECT is dependent upon VEGF signaling for induction of quiescent neural progenitor cell proliferation and is sufficient to produce an antidepressant effect [39]. Taken together, ECT is one of the strongest stimuli of hippocampal neurogenesis. It increases the rate of proliferation and maturation of new neurons in the dentate gyrus which could have a significant effect on hippocampal circuitry. ECT's therapeutic efficacy may be a result of this, proving to be an effective treatment for severe depression and other mood disorders.

Another possible treatment for neuropsychiatric disorders is deep brain stimulation (DBS). DBS is an extreme treatment for a wide range of neurological disorders such as Parkinson's disease, dystonia, chronic pain, and tremors [124]. It has proven effective in treating these disorders in initial trials [125, 126] and also in treating major depression especially in patients that do not respond to chemical antidepressants [127]. Growing evidence has focused on multiple anatomical targets in the brain with different stimulation frequencies, pulse width, and amplitude in order to obtain the ideal setting for conferring an antidepressant response (Table 4). The limited number of studies that have been conducted thus far suggests that DBS may proceed via an increase in adult neurogenesis and survival rate of mature neurons integrating into the hippocampal circuitry  (Table 4). The first evidence reported by [44] demonstrated that high frequency stimulation of the rat thalamus increased adult neurogenesis and restored experimentally suppressed neurogenesis in the dentate gyrus. One study shows that stimulation of the anterior thalamic nuclei has the effect of promoting proliferation of ANPs, similar to fluoxetine [45]. Similarly, other studies have identified specific stimulation of the entorhinal cortex, a major source of input to the hippocampus, promoting proliferation of progenitors which increase the survival rate and formation of mature neurons integrating into the hippocampal circuitry [46]. This finding was supported by increased performance in the water-maze memory tests. DBS may prove to be a significant approach in combating psychiatric disorders. However, direct involvement of adult neurogenesis conferring antidepressant action of DBS as well as its mechanism of action needs to be determined.

### 3.2. Chronic Treatment of Chemical Antidepressants

Multiple classes of antidepressants have been shown to positively influence aspects of adult hippocampal neurogenesis in a chronic time course manner (Table 5). Most selective serotonin reuptake inhibitor (SSRI) treatments are associated with a delayed onset of therapeutic efficacy consistent with the time course of maturation of newborn neurons [66]. Stimulation of neurogenesis is required for antidepressant efficacy. Studies in monkeys and rodents confirm that in conferring antidepressant action, chronic fluoxetine treatment stimulates adult hippocampal neurogenesis and mediated depressive-like behavioral effects [38, 47, 116].

The cellular basis of fluoxetine action within the neuronal differentiation cascade has been identified. Using a nestin-cyan fluorescent protein (CFP)nuc mouse line where the reporter is fused to a nuclear localization signal that allows identification and classification of early neuronal progenitors, fluoxetine was shown to increase symmetric divisions of the amplifying neuronal progenitor (ANP) cell class while not affecting division of stem-like cells in the dentate gyrus [49]. These results suggest that the cellular target for fluoxetine's therapeutic action to increase new neurons arises due to a resultant expansion of this ANP cell class. Another study by Wang et al. [48] showed that fluoxetine stimulates dendritic development of newborn neurons and neurogenesis-dependent LTP in the dentate gyrus which results in behavioral alteration induced by fluoxetine. By ablating adult neurogenesis using X-ray irradiation, they indicated that fluoxetine-induced LTP and behavior response both require adult hippocampal neurogenesis [17]. Thus, the effects of chronic fluoxetine administration on the maturation and functional properties of newborn neurons may translate into enhanced synaptic plasticity in the appropriate neural circuits, which subsequently exhibit a behavioral response to antidepressant action. Besides studying fluoxetine, various other classes of antidepressant drugs have been tested. For instance, similar results were seen in rodent administration of SSRI citalopram [50] and tricyclic antidepressant imipramine [51], although this effect was observed in the stressed condition. The mood stabilizers including lithium have been shown to significantly increase both neural progenitors and survival [128]. Although a more detailed characterization of the cellular process needs to be determined, these studies support the notion that regulation of adult neurogenesis may provide potential therapeutic targets for treatment of depression. 

What underlies the neurogenic action of antidepressants? Extrinsic factors such as BDNF and VEGF that regulate the microniche of adult neurogenesis may hold the answer. BDNF serves as a key regulator of various aspects in adult hippocampal neurogenesis including proliferation, survival, dendritic growth, maturation, and synaptic plasticity, which could make BDNF a potential mediator of the antidepressant action induced by different chemical antidepressants [129]. Emerging evidence shows that BDNF and TrkB mRNA levels in the hippocampus are dramatically induced by chronic treatment of different chemical antidepressants including fluoxetine, tranylcypromine, sertraline, desipramine, and mianserin [130]. Infusion of exogenous BDNF into the hippocampus exhibits antidepressive-like behavioral responses [120]. Further, behavioral abnormalities found in heterozygous BDNF (BDNF+/−) mice and mice lacking the TrkB are counteracted by chronic antidepressants including fluoxetine [131]. This data coupled with evidence that BDNF-signaling enhances adult neurogenesis in the dentate gyrus [132] led to the suggestion that antidepressants may represent an enhancement of neural plasticity such as adult neurogenesis and behavioral alteration, which in turn could be regulated by increasing the level of BDNF [130]. However, a direct link between adult hippocampal neurogenesis and antidepressive-like behavioral action of antidepressants through BDNF is still lacking. 

The other promising molecular target is VEGF, which shows increased mRNA levels with chronic treatment of fluoxetine and desipramine in the hippocampus [133]. VEGF is also sufficient to promote basal level of adult hippocampal neurogenesis [134] and is necessary for the antidepressant action [133]. Conversely, a significant role of VEGF is demonstrated when VEGF signaling diminishes induction of adult neurogenesis and antidepressant action in response to chronic treatment of fluoxetine [133]. Taken together, an important action of antidepressants may be to increase neurogenesis in the hippocampus through a variety of molecular mechanisms which have an impact on depressive symptoms. 

### 3.3. Exercise Therapy

Physiological stimulation in the form of exercise has been shown to stimulate cell proliferation and adult hippocampal neurogenesis [53, 55, 56, 135] and enhance the learning and memory function in both mice [57, 136] and human [137]. The plastic nature of the mammalian brain, especially neurogenesis continuing in the hippocampus well into adulthood, has allowed for exercise to exert its effects at the cellular level. This not only holds promise for brain diseases such as Alzheimer's or Parkinson's disease, but also for schizophrenia and major depression. A recent clinical trial demonstrated how exercise therapy can improve the mental health and cardiovascular fitness in patients with schizophrenia [138]. The use of exercise in treating depression has also received increased attention in recent years.

The cellular effects of running on hippocampal neurogenesis have been closely investigated in a number of notable studies (Table 6). Using BrdU labeling, one of the earlier studies showed how mice with free access to a running wheel nearly doubled the number of surviving newborn cells [53]. Another study has shown that running actually activates the quiescent radial population in the hippocampus [55]. These activated cells eventually give rise to mature neurons that are functionally integrated into the hippocampal circuitry. Hippocampal structure and function has been closely studied in relation to cognitive or mental function. Running has been shown to improve neurogenesis with a corresponding enhancement in learning and long-term potentiation [57, 136]. Physical exercise has all been implicated in the distinct encoding of spatial information. In one study, young and aged mice undergoing running exercise were compared to each other with the result that running enhanced spatial pattern separation when exercise was correlated with increased hippocampal neurogenesis [56]. The effect of exercise-induced neurogenesis has been studied in humans as well. Cerebral blood volume (CBV) maps in hippocampal formation have been generated in both exercising mice and humans [54]. Similar to mice, exercise specifically targeted the dentate gyrus CBV in humans and was correlated with increased cognitive function.

It is still unclear whether benefits from physical exercise, namely, in cognitive function, are conferred by the increase in neurogenesis. Any causal relationship between the increased neurogenesis and benefits in learning and memory is still being studied, and at least, current evidence is not decisive [139]. BDNF, as previously noted playing a role in both ECT and antidepressants, has again been suggested as a strong contender and possible mediator of the causal relationship being observed. A recent clinical trial demonstrated that exercise training increased hippocampal volume, effectively reversing age-related loss in volume [137]. This increased hippocampal volume was also associated with greater serum levels of BDNF [137]. In the review of Bekinschetein et al., a number of studies are mentioned indicating a strong causal link between BDNF and learning and memory. Physical activity has been associated with increased BDNF mRNA levels in the rat hippocampus, especially the dentate gyrus [140, 141]. Specific deletion of the BDNF-receptor TrkB reduced survival of newborn neurons, impaired neurogenesis-dependent LTP, and increased anxiety-like behavior [142]. Indeed, ablation of TrkB in neural progenitors also prevented behavioral improvements conferred by exercise [132], bolstering the role of BDNF. Other factors, namely, NMDA receptors and downstream effectors such as calcium/calmodulin protein kinase II and mitogen-activated protein kinase, could be involved in the mechanism by which BDNF effects synaptic plasticity [143]. Further evidence may ascertain the mechanism by which BDNF operates.

In an effort to treat neuropsychiatric disorders in relation to aberrant neurogenesis, it is important to study and classify important stimuli that can have a lasting effect on neurogenesis and hippocampal function. ECT, DBS, antidepressants, and physical exercise all seem to have their own effect on neurogenesis, and in combination with proper administration, they could prove vital in discovering potential treatments for psychiatric disorders that continue to disable the population.

## 4. Conclusion 

Significant progress has been made in the past decade documenting the function and regulation of adult neurogenesis. Many offer a neurobiological understanding of the role of adult neurogenesis in psychiatric disorders. These studies demonstrate how these disorders may proceed through an impairment of neural progenitor proliferation in the hippocampus, and how an ablation of neurogenesis may predispose an animal to depressive-like behaviors. A pathophysiologically reliable animal model is, however, still required to confirm data across the cellular and behavioral spectrums. Further, genetic manipulations of susceptibility genes in loss-of-function transgenic models may be used to rescue cognitive deficits and confirm their roles via cellular and behavioral studies. These may bolster a causal link between adult neurogenesis and the disorder.

However, with our current knowledge, several questions remain to be answered. We do not yet have a clear understanding of how external stimuli in current treatments mediate the induction and function of factors in the neurogenic niche that stimulate adult hippocampal neurogenesis. We also do not know how the regulation of neurogenic niche via niche signals and neurotrophic factors may be altered by X-ray irradiation which is used to ablate and study neurogenesis. The role of BDNF has been evidenced in ECT, DBS, antidepressants, and exercise therapy. Going forward, its downstream effects would need to be studied to determine how BDNF mediates synaptic plasticity. Further, in the case of relatively new treatments such as DBS, conclusive evidence of its antidepressant effect and mechanism of action will bolster the significant role it could play in treating psychiatric disorders. Finally, we are yet to obtain a conclusive causal relationship between adult neurogenesis and depression, as well as adult neurogenesis and cognitive function or learning and memory. How drug abuse may alter these relationships especially in the maturation and integration of newborn neurons in the hippocampus will be useful. Current evidence when coupled to such a characterization may provide evidence of the cellular mechanism at play in many widely used treatments.

Adult neurogenesis is important in synaptic plasticity with overarching roles in memory, learning, and mood. Such roles have been established via a plethora of studies which show that the process is dynamically regulated and demonstrates striking structural plasticity in response to internal and environmental cues. It is strongly regulated by stress signals, and stimulated by antidepressants, which may serve to potentially alter the hippocampal circuitry. Current evidence indicates a significant role for adult neurogenesis in the neurological impairments of psychiatric disorders. At this critical juncture, it is important we distinguish whether adult neurogenesis is causally involved in the etiology or plays a significant role in ameliorating the disease state symptoms. A clear understanding of this relationship in regard to the pathogenesis of the disease will be invaluable in aiding our understanding of the causes at play, and how effective treatments may be designed to alleviate the symptoms.

## Figures and Tables

**Table 1:** Select animal studies investigating the effect of depression on adult hippocampal neurogenesis and behavior.

Species	Stress paradigm	Experimental approach	Effects on neurogenesis	Effects on behavior	References
Physical stress					

Sprague-Dawley rat	Acute restraint stress: either 2 or 6hours. Chronic restraint stress: 6 hours daily for 21 days. Repeated restraint stress: 6 hours daily for 42 days.	Proliferation: BrdU (1 × 200 mg/kg, ip) at 2 hours pulsing chase Survival: BrdU (100 mg/kg, ip) daily for 4 days and analysis on 12 days to 3 weeks periods after BrdU injection.	Acute stress: no effect on cell proliferation. Chronic stress: Decreased cell proliferation, slightly decreased survival rate with no statistical significance. Repeated stress: decreased survival rate.	Not studied (NS)	[11]

Sprague-Dawley rat	60 inescapable foot shocks	Proliferation: BrdU (1 × 100 mg/kg, ip) at 2 hours pulsing chase. Survival: BrdU (100 mg/kg, ip) on day 9 and analysis 28 days later.	Decreased cell proliferation. No change on survival rate and differentiation.	NS	[12]

ICR mouse	Chronic restraint stress: 6 hours per day for four weeks.	Proliferation: Ki67 immunostaining. Survival: BrdU (3 × 75 mg/kg, ip) at 6-hour intervals and 24 hours before exposure to chronic restraint stress.	Decreased cell proliferation and survival rate.	Impaired context dependent memory.	[13]

Wistar rat	Olfactory bulbectomy (OB).	Proliferation: BrdU (1 × 40 mg/kg, ip) at 2 hours pulsing chase. Survival: BrdU (3 × 200 mg/kg, ip) at 2-hour intervals and analysis 4 weeks after last administration.	Decreased cell proliferation and neuronal differentiation.	Impaired context-dependent memory.	[14]

Social stress					

Sprague-Dawley rat	Psychosocial stress by introducing intruder animal.	Proliferation: IdU (4 × 57.5 mg/kg, ip) at 12-hour intervals. IdU at 3-day pulsing chase after the first administration. Short-term survival: BrdU (6 × 50 mg/kg, ip), 12-hour intervals. BrdU at 10-day pulsing chase after first administration.	No change on cell proliferation, but decreased short-term survival rate.	NS	[15]

Prairie vole	Social isolation: single-housed for 6 weeks.	Proliferation: Ki67 immunostaining. Survival: BrdU (100 mg/kg, ip) daily for fourteen consecutive days and then 6-week pulsing chase after last administration.	Decreased cell proliferation and neuronal maturation.	Higher levels of anxiety-like and depression-like behaviors	[16]

Wistar rat	Social defeat stress daily for 18 days	Proliferation: BrdU (200 mg/kg, ip) at 24-hour pulsing chase. Survival: BrdU (200 mg/kg, ip) two times per day for 2 days.	Decreased cell proliferation and survival rate.	NS	[17]

**Table 2:** Select animal studies investigating the effect of schizophrenia on adult hippocampal neurogenesis and behavior.

Species	Susceptibility genes (animal model)	Experimental approach	Effects on neurogenesis	Effects on behavior	References
C57BL6/Jmouse	DISC1	Knockdown of *Disc1* via retroviral-mediated shRNA approach.	Enhanced dendritic outgrowth, soma hypertrophy, overextended migration, accelerated synapse formation of newborn granule neurons.	NS	[18]

C57BL6/Jmouse	DISC1	Knockdown of *Disc1* via retroviral-mediated shRNA approach.	Mistargeted axonal mossy fibers and presynaptic differentiation of newborn granule neuron.	NS	[19]

C57BL6/Jmouse	DISC1	Knockdown of *Disc1* via lentiviral-mediated shRNA approach. Proliferation: BrdU (1 × 40 mg/kg, ip) at 2 hours pulsing chase.	Decreased cell proliferation	Hyperlocomotion in a novel environment. Depressive-like behavior.	[20]

129/SvEv	DISC1 (Disc1^Tm1Kara^ mutant)	BrdU (50 mg/kg, ip) for 12 days.	Decreased neural progenitors and immature neurons. Misoriented apical dendrites on immature neurons.	Selective impairment in working memory	[21]

129/SvEv C57BL/6J mouse	NPAS3 *Npas3* null mouse	Proliferation: BrdU daily (50 mg/kg, ip) for 12 days and analysis on day 13	Decreased cell proliferation in *Npas3* null mouse.	Locomotor hyperactivity, subtle gait defects, impairment of prepulse inhibition of acoustic startle, deficit in recognition memory, and altered anxiety-related responses.	[22, 23]

C57BL6/Jmouse	SREB2 Overexpression of SREB2 transgenic mouse (Tg) SREB2 null mouse	Proliferation: BrdU (3 × 100 mg/kg, ip) interval 2 hours apart and analysis on Day 3 after BrdU injection. Survival: Three BrdU pulses (100 mg/kg, ip) at 2-hour intervals. Analysis on Day 30 following BrdU injection.	SREB2 Tg: decreased cell proliferation and neuronal maturation. Dendritic morphology deficits in newborn granule neurons. SREB2 null mouse: no change in cell proliferation, but increased neuronal survival rate.	SREB2 Tg: decreased discrimination in the spatial pattern separation. SREB2 null mouse: enhanced discrimination in the spatial pattern separation.	[24]

C57BL6/Jmouse	Prenatal injection of synthetic double-stranded RNA polyriboinosinic polyribocytidylic acid (PolyI:C) into mice	BrdU (100 uL/20 g, ip) daily for 3 days and analysis 4weeks later.	Decreased neural progenitors.	Behavioral deficits in the open field test and prepulse inhibition of the startle response.	[25, 26]

**Table 3:** Select animal studies investigating the effect of drug addiction on adult hippocampal neurogenesis.

Species	Drug treatment	Experimental approach	Effect on neurogenesis	References
Cocaine				

Cocaine/rat (Sprague-Dawley)	Self-administration (0.5 mg/kg infusion, iv) for 3 weeks.	Proliferation: BrdU (150 mg/kg, ip) once at 2-hours pulsing chase after the last self-administration session. Survival: BrdU at four-week pulsing chase after last self-administration session.	Cell proliferation: decreased number of BrdU+ cells. Survival: no change.	[27]

Cocaine/rat (Sprague-Dawley)	20 mg/kg (ip) for either 1 or 14 days	BrdU once (100 mg/kg, ip) at 2-hour pulsing chase on either 1 or 14 days during cocaine exposure.	One-day cocaine injection: no change in cell proliferation Fourteen-day cocaine injection: decreased cell proliferation	[28, 29]

Sprague-Dawley rat	Self-administration (0.5 mg/kg/20 s or 1.5 mg/kg/20 s infusion, iv) for 2 weeks.	Proliferation: BrdU (3 × 50 mg/kg, ip) at 4-hour intervals; analysis twenty-four hours after last BrdU injection. Survival: BrdU (3 × 50 mg/kg, ip) at 4-hour intervals; analysis 4 weeks after last BrdU injection.	Proliferation: decreased number of BrdU+ cells. Survival: decreased neurogenesis.	[30]

Cocaine/rat (Wistar)	20 mg/kg (ip) once daily for 8 days.	BrdU once (40 mg/kg, ip) at 2-hour pulsing chase on final day of cocaine exposure.	Decreased number of BrdU+ cells for cell proliferation. Decreased number of Ki67+ cells with no statistical significance.	[31]

Cocaine/rat (Wistar)	20 mg/kg (ip) daily for 24 days.	BrdU (40 mg/kg, ip) daily for 7 days during the first 7days of cocaine exposure and Ki67 immunostaining.	Decreased number of Ki67+ cells for cell proliferation. No effect on survival.	[31]

Cocaine/rat (Wistar)	20 mg/kg (ip) daily for either 8 or 24 days.	BrdU (2 × 140mg/kg, ip) at 6-hour intervals and 24hours before receiving cocaine exposure.	Both 8-and 24-day cocaine injection: no effect on maturation	[31]

Alcohol				

Sprague-Dawley rat	Acute: EtOH (5 g/kg) by gavage. Chronic: EtOH (5 g/kg) via intragastric catheter on every 8 hours for 4 days.	Proliferation: Acute: BrdU (2 × 100 mg/kg, ip) at 4 hours 15 minutes and 2 hours and 15 minutes pulsing chase. Chronic: BrdU (4 × 100 mg/kg, ip) daily for 4-day binge EtOH Survival: Acute: BrdU (2 × 100 mg/kg, ip) at 4-week pulsing chase. Chronic: BrdU (4 × 50 mg/kg, ip) at 4-week pulsing chase.	Acute: both proliferation and neuronal maturation decreased. Chronic: both proliferation and neuronal maturation decreased.	[32]

Sprague-Dawley rat	EtOH (final concentration, 6.4% vol/vol) by gavage for 6 weeks.	Proliferation: BrdU (7 × 40 mg/kg, ip) at 2-hour intervals; animals were sacrificed 1 hour after last BrdU injection. Survival: BrdU (40 mg/kg, ip) once daily for first 10 days of 6 weeks EtOH binge; animals were sacrificed 32 days after the last dose of EtOH.	Proliferation: decreased cell proliferation. Maturation and survival: decreased neuronal maturation.	[33]

Wistar rat	Alcohol nondependent: Self-administered alcohol for 3 weeks and exposed air for 10 weeks. Alcohol dependent: Self-administered alcohol for 3 weeks and exposed to intermittent alcohol vapors for 10 weeks.	At week 6, BrdU (150 mg/kg, ip) at 4-week pulsing chase. Immunostaining: Fluoro-Jade C: cell death neuronal degeneration AC-3: apoptotic cells. Ki-67: proliferating cells. DCX: immature neurons. NeuN: mature neurons.	Nondependent drinking: increased cell death and decreased cell proliferation, immature neurons and survival. Dependent drinking: increased cell death and decreased cell proliferation, immature neurons and survival.	[34]

C57BL/6J mouse	EtOH by gavage for 28 days and abstinence for either 1 or 14 days.	BrdU (3 × 300 mg/kg, ip) at 4 weeks pulsing chase. Immunostaining: DCX: immature neurons PCNA: proliferating cell marker Behavior tests: open-field locomotor activity and forced swimming test.	Abstinence following alcohol drinking caused: (i) no change in survival of neuronal progenitor cells. (ii) decreased proliferative activity of progenitor cells. (iii) decreased neurogenesis. (iv) increased depressive-like behavior. (v) transiently (1 day abstinence) increased locomotor activity.	[35]

Rhesus Monkey	Alcohol induction: EtOH (1%) by gavage for 40 sessions. Alcohol self-administration: EtOH (6%) by gavage for 200 sessions over 11 months. Alcohol abstinence: abstinence for 2–2.5 months.	Immunostaining: Fluoro-Jade C: cell death neuronal degeneration AC-3: apoptotic cells. Ki-67: proliferating cells. GFAP: radial glia-like stem cell and astrocyte marker. SOX2: amplifying neural progenitor cell marker. NeuroD1: immature neurons. PSA-NCAM: mature neurons.	Abstinence following alcohol drinking caused: (i) decreased neurogenesis, (ii) decreased proliferative activity of progenitor cells in initial phases of neuronal development.	[36]

**Table 4:** Select animal studies investigating the effect of electroconvulsive therapy (ECT) and deep brain stimulation (DBS) on adult hippocampal neurogenesis.

Species	ECT treatment	Experimental approach	Effects on neurogenesis	Molecular target	References
Electroconvulsive therapy					

Wistar rat	Single ECT	BrdU (2 × 37.5 mg/kg, ip) at 12-hour intervals, starting at 0, 3, 5, 7, and 9 days after ECT and analysis 48 hours after the last injection of BrdU.	Increased cell proliferation.	NS	[37]

Sprague-Dawley rat	Chronic ECT: once daily for 10 days	Proliferation: BrdU (4 × 45 mg/kg, ip) with 2-hour intervals, on 4 days after ECT and analysis at 24 hours after last BrdU injection. Survival: BrdU (4 × 45 mg/kg, ip) with 2-hour intervals before ECT treatment and analysis at 28 days after last BrdU injection.	Increased cell proliferation and survival rate.	NS	[38]

Sprague-Dawley rat	Single ECT Chronic ECT: once daily for 7 days	BrdU (150 mg/kg, ip) at 2 or 24 hours pulsing chase.	2-hour BrdU pulse chase after single ECT: increased quiescent neural progenitors (QNPs) and slightly increased amplifying neural progenitors (ANPs) with no statistical significance. At 24-hour BrdU pulse chase after single and chronic ECT: increased QNPs and ANPs.	VEGF	[39]

Wistar rat	ECT every second day for a course of 8 seizures	Starting with seizure number 5, BrdU (1 × 50 mg/kg, ip) at 2-or 3-hour intervals following each ECT treatment. Injections were also given at the same time during the 3 days between treatments, resulting in a total of seven injections.	Increased maturation of newborn neurons.	NS	[40]

Sprague-Dawley rat	ECT two times	NIT-GFP retroviral injection after ECT treatment.	Increased mushroom spine density on newborn granule neuron dendrites.	NS	[41]

C57BL6/J mouse	Single ECT	BrdU (1 × 200 mg/kg, ip) at 2-hour pulsing chase. GFP retroviral injection.	Increased cell proliferation. Increased total dendritic length and dendritic complexity.	Gadd45b	[42]

Bonnet monkey	ECT: three times a week for four weeks	Proliferation: BrdU (100 mg/kg, iv) daily for 4 days. Survival: BrdU (100 mg/kg, iv) daily for 4 days and analysis at 4 weeks after BrdU injection.	Increased cell proliferation and neuronal maturation.	BCL2	[43]

Deep Brain Stimulation					

Sprague-Dawley rat	Anterior thalamic nucleus was stimulated at 2.5 V, 90 *μ*se of pulse width, and variable frequencies (10, 50, 130 Hz) for an hour.	BrdU (4 × 50 mg/kg, ip) with 3-hour interval and analysis at 24 hours or 4 weeks after last BrdU injection.	Increased cell proliferation. No change in neuronal maturation	NS	[44]

C57BL/6 mouse and nestin-CFPnuc transgenic mouse	Anterior Thalamic Nucleus was stimulated at 2.5 V, 90 *μ*s of pulse width, and frequencies 10 Hz (low) and 130 Hz (high) for an hour.	BrdU (3 × 50 mg/kg, ip) with 3-hour intervals and analysis at 24 hours or 30 days after last BrdU injection.	Increased cell proliferation in high frequency but not low frequency. Increased proliferation of amplifying neural progenitors (ANPs), but no change in quiescent neural progenitors (QNPs) at high frequency. Increased neuronal maturation at high frequency but not low frequency.	NS	[45]

Mixed genetic background between C57BL/6NTacfBr and 129Svev mice	Entorhinal cortex at at 0–500 *μ*A current, for 30–120 minutes, at 90 *μ*s of pulse width and 130 Hz frequency.	Proliferation: BrdU (1 × 200 mg/kg, ip) injection on day 1, 3, 5, or 7 after stimulation and analysis at 24 hours after BrdU injection. Neuronal maturation: IdU (57 mg/kg, ip) injections during the period of stimulation-induced increased proliferation (postoperative days 3–5), CldU (42.5 mg/kg, i.p.) injections during a similar period of baseline proliferation (postoperative days 7–9), and analysis about 10 weeks later. Survival: BrdU daily (100 mg/kg, ip) with 8-hour intervals for 3 days on 1, 10, or 30 days before stimulation and postoperative analysis 3 weeks later. Dendritic development: GFP-retroviral injection.	Increased cell proliferation after 60 or 120 minutes of stimulation and at 50, 250, and 500 *μ*A. No change in fate determination of newborn cells. Increased dendritic length of newborn granule neurons but no change in nodes per neuron and dendritic spine size.	Improved spatial memory formation measured by water maze.	[46]

**Table 5:** Select animal studies investigating the effect of chemical antidepressants on adult hippocampal neurogenesis and animal behavior.

Species	Antidepressant treatment	Experimental paradigm	Effect on neurogenesis	Effects on behavior	References
129/SvEv	Fluoxetine (SSRI): 10 mg/kg/day via drinking water for 28 days	Proliferation: BrdU (4 × 75 mg/kg, ip) at 2-hour intervals and analysis at 24 hours after last BrdU injection. Survival: BrdU (4 × 75 mg/kg, ip) at 2-hour intervals and analysis at 28 days after last BrdU injection.	Increased cell proliferation and neuronal maturation.	Less depressive-like behavior in novelty suppressed feeding test.	[47]

Sprague Dawley rat	Fluoxetine (SSRI): 5 mg/kg (ip) 1, 5, 14, 28 d. Tranylcypromine: 7 × 7.5 mg/kg (ip) daily, then 14 × 10 mg/kg (ip) daily. Reboxetine: 20 mg/kg, 2x per day for 21 d	Proliferation: BrdU (4 × 75 mg/kg, ip) at 2-hour intervals, then 24-hour pulsing-chase. Survival: BrdU (4 × 75 mg/kg, ip) at 2-hour intervals, then 28-day pulsing chase.	Fluoxetine (SSRI): increased cell proliferation and neuronal maturation. Tranylcypromine: increased cell proliferation Reboxetine: increased cell proliferation.	NS	[38]

129/SvEv	Fluoxetine (SSRI): 18 mg/kg daily by gavage for behavior test and via drinking water for all experiments for 5 days (subchronic) or 28 days (chronic).	Proliferation: BrdU (1 × 150 mg/kg, ip) at 2-hour pulsing chase. Dendritic maturation, survival, and neuronal maturation: BrdU (4 × 75 mg/kg, ip over 8 hours) on day 0, started fluoxetine treatment on day 1, and analysis on day 21 by BrdU and DCX coimmunostaining.	Increased cell proliferation in chronic treatment of fluoxetine. No change in number of DCX+ immature neurons. Increased dendritic length and dendritic complexity in chronic treatment of fluoxetine, but not subchronic treatment measured by BrdU+ DCX+ colabeled neurons. Increased survival rate and neuronal maturation in chronic treatment of fluoxetine.	Less depressive-like behavior by chronic fluoxetine treatment (but not subchronic) in novelty suppressed feeding test.	[48]

Nestin-CFPnuc mouse	Fluoxetine (SSRI): 10 mg/kg (ip) daily for 15 days.	Proliferation: BrdU (1 × 150 mg/kg, ip) and analysis 1 day later. Survival: BrdU (1 × 150 mg/kg, ip) and analysis 30 days later.	Increased proliferation of amplifying neural progenitors (ANPs), but no change in quiescent neural progenitors (QNPs). Increased survival rate.	NS	[49]

Wistar rat	Escitalopram (SSRI): 5 mg/kg (ip) for 4 weeks.	BrdU (4 × 100 mg/kg, ip) with 2-hour interval during one day and analysis at 16 hours after the last BrdU injection.	Increased cell proliferation in chronic treatment of escitalopram under mild stress condition, but no change under the normal condition.	Chronic treatment of escitalopram shows recovery from anhedonic-like behavior caused by the mild stress condition.	[50]

BALB/c mouse	Fluoxetine (SSRI): 10 mg/kg (ip) daily for 28 days, Imipramine (TCA): 20 mg/kg (ip) daily for 28 days.	BrdU (4 × 75 mg/kg, ip) with 2 hours interval and analysis at 24 hours after last BrdU injection.	Increased cell proliferation in chronic treatment of fluoxetine and imipramine under unpredictable chronic mild stress.	Chronic treatment of fluoxetine and imipramine shows recovery from depressive-like behavior caused by unpredictable chronic mild stress condition.	[51]

Wistar rat	Lithium chloride: 2.5 mEq/kg (ip) for 14 days.	BrdU (50 mg/kg, ip) daily during last 3 days of experimental period	Increased proliferation, neuronal maturation and glial maturation of neural progenitors under normal conditions. Lithium treatment shows recovery from neurogenesis deficits under unpredictable chronic mild stress condition.	Lithium treatment shows less depressive-like behavior under normal conditions. Lithium treatment shows recovery from depressive-like behavior caused by unpredictable chronic mild stress condition.	[52]

*SSRI: Selective serotonin reuptake inhibitor; TCA: Tricyclic Antidepressants; DCX: doublecortin (immature neuronal marker) 13.

**Table 6:** Select animal studies investigating the effect of exercise on adult hippocampal neurogenesis.

Species	Type of exercise	Experimental paradigm	Effect on neurogenesis	Effects on behavior	References
C57BL/6 mouse	Learners: 2 trials of Morris water maze training per day over 30 days. (with platform). Swimmers: 2 trials of morris water maze training per day over 30 days (without platform). Runners: 1 running wheel for 3-4 mice in rat cage.	Proliferation: BrdU (12 × 50 mg/kg, ip) daily for 12 days, then 24-hour pulsing chase. Survival: BrdU (12 × 50 mg/kg, ip) daily for 12 days, then 40-week pulsing chase.	Learners: decreased cell proliferation and survival of newly generated neurons. Swimmers: decreased cell proliferation and survival of newly generated neurons Runners: increased cell proliferation and survival of newly generated neurons.	NS	[53]

C57BL/6 mouse	Voluntary running.	MRI scan at weeks 0, 2, 4, and 6. Survival: BrdU (7 × 60 mg/kg, ip) during second week of experiment, then 4-weeks pulsing chase.	Exercise: (i) increased cerebral blood volume specifically at dentate gyrus. (ii) increased neuronal maturation.	NS	[54]

Hes5::GFP mouse	Voluntary running.	BrdU (50 mg/kg, ip) either once or 3 consecutive times (2-hour interval); then 2 hour or 5-day pulsing chase.	Exercise activated QNPs in dentate gyrus.	NS	[55]

C57/BL6 mouse	Voluntary running.	Spatial pattern separation. BrdU (5 × 50 mg/kg, ip) daily for 5 days, then 10-week pulsing chase.	Exercise increased neuronal maturation.	Exercise increased spatial pattern separation.	[56]

C57/BL6 mouse	Voluntary running.	Spatial learning: Morris water maze task day 35–39. Proliferation: BrdU (7 × 50 mg/kg, ip) daily for 7 days, then 24-hour pulsing chase. Neuronal morphology analysis: 4-week dpi GFP retrovirus. Blood vessel size analysis: Lectin staining	Proliferation: increased proliferation in both young and old mice. Neuronal morphology analysis: increased dendritic length and branches in young runners. increased blood vessel size in young runners only.	Exercise enhanced spatial learning in both young and old mice.	[57]
